# Effect of internet-based cognitive behaviour therapy among women with negative birth experiences on mental health and quality of life - a randomized controlled trial

**DOI:** 10.1186/s12884-022-05168-y

**Published:** 2022-11-12

**Authors:** Josefin Sjömark, Agneta Skoog Svanberg, Margareta Larsson, Frida Viirman, Inger Sundström Poromaa, Alkistis Skalkidou, Maria Jonsson, Thomas Parling

**Affiliations:** 1 Department of Women’s and Children’s Health, Uppsala University, Dag Hammarskölds väg 14 B, SE-751 85 Uppsala, Sweden; 2 Department of Clinical Neuroscience, Centre for Psychiatry Research, Karolinska Institutet, & Stockholm Health Care Services, Region Stockholm, Stockholm, Sweden

**Keywords:** Cognitive behavioural therapy, Internet intervention, Internet-based CBT, Negative birth experience, Posttraumatic stress disorder, PTSD following childbirth

## Abstract

**Background:**

Giving birth is often a positive experience, but 7–44% have negative experiences and about 4% develop posttraumatic stress disorder following childbirth (PTSD FC). This randomized controlled trial (RCT) investigated the effect of internet-based cognitive behaviour therapy (iCBT) for women with negative birth experiences and/or at risk for PTSD FC.

**Methods:**

This was a superiority nonblinded multicentre RCT comparing iCBT combined with treatment as usual (TAU) with TAU only. Data were collected through questionnaires at baseline, at 6 weeks, 14 weeks and 1 year after randomization. The RCT was conducted at four delivery clinics in Sweden and participants were recruited from September 2013 until March 2018. Women who rated their childbirth experience as negative on a Likert scale, and/or had an immediate caesarean section or a haemorrhage of > 2000 ml were eligible. Primary outcomes were symptoms of posttraumatic stress (Traumatic Event Scale, TES) and symptoms of depression (Edinburgh Postnatal Depression Scale, EPDS). Secondary outcomes were satisfaction with life (Satisfaction With Life Scale, SWLS) and coping (Ways of Coping Questionnaire, WCQ).

**Results:**

Out of 1810 eligible women, 266 women were randomised to iCBT+TAU (*n* = 132) or to TAU (*n* = 134). In the iCBT+TAU group 59 (45%) completed the treatment. ICBT+TAU did not reduce PTSD FC at 6 weeks, at 14 weeks, or at 1 year follow-up compared with TAU, according to the TES. Both the ITT and completer analyses showed significant time and quadratic time effects due to reduction of symptoms in both groups on the TES (re-experience subscale) and on the EPDS, and significant time effect on the self-controlling subscale of the WCQ (which increased over time). There was also a significant main effect of group on the SWLS where the TAU group showed higher initial satisfaction with life. Exploratory subgroup analyses (negative birth experience, immediate caesarean section, or severe haemorrhage) showed significant time effects among participants with negative birth experience on re-experience, arousal symptoms and depressive symptoms.

**Conclusions:**

The ICBT intervention did not show superiority as both groups showed similar beneficial trajectories on several outcomes up to 1 year follow-up. This intervention for women with negative birth experiences and/or at risk for PTSD FC was feasible; however, the study suffered from significant drop out rate. Future studies with more narrow inclusion criteria and possibly a modified intervention are warranted.

**Trial registration:**

ISRCTN39318241. Date for registration 12/01/2017.

## Introduction

### Background

Childbirth is a major life event and can be associated with both positive and negative psychological responses [1, 2]. In the existing literature, there is no consistent definition of a negative birth experience and no suggested systematic method to assess birth-related psychological trauma. A negative birth experience is often the result of an obstetric complication, but uncomplicated normal births can also lead to a negative experience [3]. The birth experience is the woman’s subjective assessment; it is multidimensional and can be influenced by several factors such as mode of delivery, fear for self and / or the infant, loss of control, cultural and environmental factors [3, 4].

In Sweden, approximately 115,000 women give birth every year [5]. In a systematic review, the prevalence of negative birth experience ranges between 7 and 44% [6] while in a Swedish study, a prevalence of 9.6% is suggested [7]. Emergency caesarean section, extreme pain, obstetric anal sphincter injury and oxytocin augmentation have been associated with negative birth experience, together with lack of support from partner or staff [8]. Distress from a negative birth experience increases the risk for mood disorders in the postpartum period [9] and can lead to the development of Posttraumatic Stress Disorder Following Childbirth (PTSD FC) [10–15]. According to DSM-IV [16], symptoms of PTSD are grouped into three clusters; 1) re-experiencing the traumatic event, 2) persistent avoidance of reminders of the event and, 3) increased arousal e.g. hypervigilance or difficulty concentrating. The symptoms must have been present for at least a month and cause significant distress and impairment in occupational and social functioning. The mean prevalence of PTSD FC is 3–4% for women in community samples and 15.7–18.5% in high-risk samples (e.g. women who had emergency caesarean sections, severe fear of birth, a history of sexual/physical violence, babies born with very low birth weight or preterm babies) [15, 17]. PTSD FC can have lifelong impact and women with PTSD FC often report negative changes in their psychological wellbeing and have more psychiatric health problems, especially depressive symptoms, compared to women without a traumatic experience [18, 19]. The psychological state of the mother can also negatively affect her social interactions, such as the partner- relationship and bonding to the child [3, 18, 20–23].

Cognitive Behaviour Therapy (CBT) is the treatment of choice for most anxiety disorders [24, 25]. It is also known that trauma-focused CBT (TF CBT) can be applied for perinatal women [26]. In Sweden there are no specific treatment recommendations for PTSD FC, but in some other western countries the methods of treatment for general PTSD are also recommended for treatment of PTSD FC [26, 27]. Few studies have investigated psychological interventions for women with PTSD FC. In a systematic review and meta-analysis on the effectiveness of trauma-focused psychological therapies for PTSD FC [28], including ten RCTs, it was suggested that trauma-focused interventions are effective for reducing symptoms of PTSD FC up to 6 months postpartum but there was no evidence that trauma-focused interventions can improve women’s recovery from clinically significant PTSD FC.

Only one RCT has evaluated the effect of internet-based CBT (iCBT) for PTSD FC and found that iCBT had positive effect on both depressive symptoms and anxiety [29]. ICBT has many advantages over face-to-face CBT. It gives the patients flexibility to work with the material at any time they prefer and it reaches the patient independently of geographical location [30]. ICBT also has the advantage of greater flexibility and higher anonymity for the patient. In summary, both negative birth experience and PTSD FC can affect the wellbeing of mother and child to a large extent, and at the same time, few RCTs have investigated the efficacy of iCBT for this population.

The primary aim of this study was to evaluate the efficacy of an internet-based self-help program based on CBT compared to treatment as usual (TAU) for women with negative birth experience and/or PTSD FC in a randomized controlled trial. We hypothesized that adding iCBT to TAU will be more effective than TAU alone in reducing symptoms of posttraumatic stress and depressive symptoms. We also presumed that iCBT would increase participants’ quality of life and positively affect their coping abilities.

## Method

### Design and setting

This study was a superiority non-blinded multicentre RCT comparing iCBT+TAU with TAU [31]. The study is associated with the U-CARE program/ eService. The U-CARE portal (http://www.u-care.uu.se) was used for data collection and the internet intervention. Recruitment was conducted at four delivery clinics in Sweden; three university hospitals and one regional hospital with 2700, 2800, 3000 and 4000 annual births.

### Participants

Women were recruited between September 2013 and March 2018. Before discharge from the hospital, women rated their overall childbirth experience on a Likert-scale (range; 0–10 with lower numbers corresponding to worse experience). Inclusion criteria were a rating ≤ 5 on the Likert scale and/or exposure to an immediate caesarean section and/or a severe haemorrhage (≥2000 ml) following childbirth. Exclusion criteria were (1) age under 18 years, (2) no adequate access or ability to use the internet, (3) no adequate ability to speak, read or write Swedish, (4) severe mental illness based on the participants statement, (5) stillbirth and neonatal death or (6) other on-going psychological treatment. The final sample consisted of 266 participants (iCBT+TAU *n* = 132 and TAU *n* = 134) (Table 1).

**Table 1:** Characteristics of the participants

	Total *n* = 266	ICBT+TAU *n* = 132	TAU *n* = 134	Statistic
	*M* (*SD*)	*M* (*SD*)	*M* (*SD*)	
Age	31.76 (4.41)	31.62 (4.23)	31.90 (4.55)	n.s
Overall childbirth experience	3.56 (1.95) *n* = 221	3.57 (2.13) *n* = 112	3.56 (1.75) *n* = 109	n.s
	*N*	*N*	*N*	
Parity; 0/1/≥2	185/50/31	94/24/14	91/26/17	n.s
Education: Elementary school / High school / University	4/67/195	2/39/91	2/28/104	n.s
Relationship status: Married or cohabit / Single or other	260/6	130/2	130/4	n.s
Occupation: Working / Unemployed / Student/Sick leave	221/12/21/12	104/8/13/7	117/4/8/5	n.s
Mode of delivery: Vaginal delivery / Emergency CS / Immediate CS / Elective CS / Vacuum assisted delivery	97/40/50/10/44	47/20/24/5/23	50/20/26/5/21	n.s
Inclusion: negative rating of birth experience / Immediate CS / severe haemorrhage	195/39/32	94/20/18	101/19/14	n.s
Severe haemorrhage: ≥2000 ml / < 2000 ml	33/227	19/112	14/115	n.s
Country of birth				n.s
Sweden / Foreign born	244/22	122/10	122/12	
Counseling for fear of childbirth; yes/no	34/232	12/120	22/112	n.s

*CS* Caesarean section

### Procedure

**Fig. 1:**
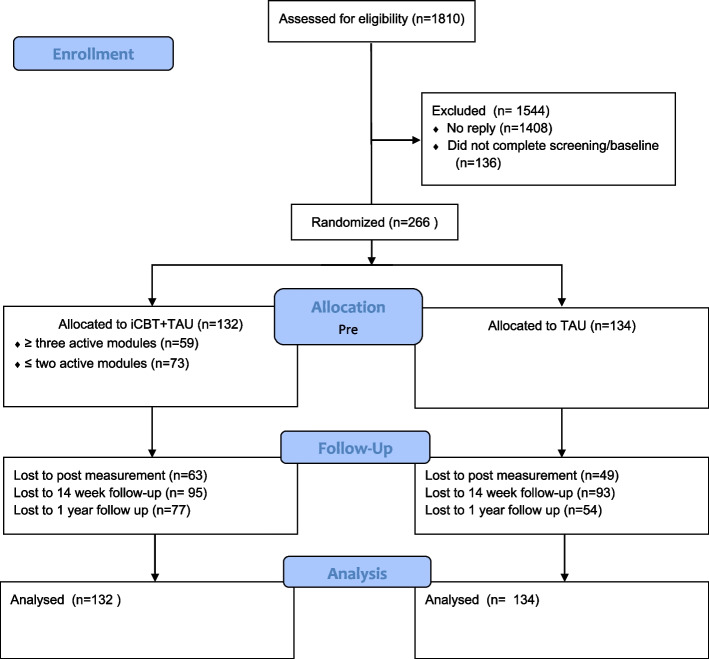
Flowchart of participants and assessments in the study

Eligible women (*n* = 1810) were contacted via telephone and informed about the study around 8 weeks postpartum (Fig. 1). Interested women (*n* = 936) and those who did not answer the telephone calls (*n* = 437) had study information and a consent form sent home by post. The partners of the women were also invited to participate. Women, who gave written consent (*n* = 402) were provided with login details to the online study site and were asked to log on and fill out the pre-intervention questionnaire. Those who completed the baseline assessment were randomly allocated to either iCBT+TAU (*n* = 132) or TAU (*n* = 134) (the partners were allocated together with the women). The 1:1 randomization procedure was automatic on the U-CARE platform with no involvement of researchers. The intervention was delivered through a secure internet-based platform (The U-CARE portal), using double verification for log in. Inactive participants were reminded via messages in the portal and/or via SMS text messages. All participants in the study were treated according to local guidelines by the health care system, regardless of treatment allocation.

### Tau

TAU included conventional support according to existing practice at the departments of the participating hospitals. The intention of all study centres was to offer women post-partum counselling if needed during their hospital stay. However, the content of TAU differed widely between the hospitals, some women were never offered counselling or planned follow up, while for others, TAU, could include follow-up with a physician and/or counselling with a specially trained midwife or routine follow-up at the midwifery antenatal care clinic.

### ICBT + tau

The iCBT intervention developed for this study was adapted to meet the needs of women with negative birth experiences. The aim of the intervention was to help them better understand their psychological reactions and find new ways to cope with dysfunctional thoughts and behaviors. The material was in Swedish, and participants could listen to an audio-visual presentation as well as view videos covering psychoeducation or choose to download written material. The intervention consisted of two parts (iCBT part 1 and iCBT part 2). ICBT part 1 included six steps (Table 2). The participants were recommended to complete one step per week and each step included homework assignments. The participants could contact the study-specific psychologist on demand. Participants who completed iCBT part 1 and fulfilled the criteria for PTSD (according to the Traumatic Event Scale, described below in *measurement* section) were offered iCBT part 2 (Table 2). ICBT part 2 was based on recommendations and evidence-based treatment for PTSD [32] and consisted of eight steps. The content was structured, individualized, and included weekly therapeutic support via e-mail.

**Table 2:** The components of the internet-based cognitive behavioural therapy (iCBT) intervention, part 1 (six steps) and part 2 (eight steps)

Part	Content
Part 1	
1	Information, psychoeducation and breathing retraining
2	Vignettes, information of common symptoms, fear, and avoidance
3	Depressive symptoms, significance of relations and information about reflective listening
4	Exposure, talking about the childbirth with others
5	Managing anxiety and depressive symptoms, information about psychological health, life-values, and recovery
6	Summary, repetition, and relapse prevention
Part 2	
1	Introduction to treatment part 2, psychoeducation regarding PTSD
2	Identify & recognize symptoms, breathing retraining (continued through treatment)
3	In vivo-exposure (continued through treatment)
4	Refined in vivo-exposure + intro to expressive writing
5	Expressive writing, imaginal exposure
6	Refined imaginal exposure
7	Finding hot-spots, recovery
8	Summary, maintaining progress, relapse prevention

### Measurements

Socio-demographic data (e.g. age, level of education and relationship status) and information about births were collected at baseline via medical records and self-reports. The study had two primary outcome measures (symptoms of posttraumatic stress assessed with The Traumatic Event Scale, TES, and depressive symptoms assessed with Edinburgh Postnatal Depression Scale, EPDS), and two secondary outcome measures (quality of life assessed with The Satisfaction with Life Scale, SWLS and ability to cope assessed with The Ways of coping questionnaire, WCQ).

TES measures post-traumatic stress symptoms related to childbirth; the scale is a self-assessed measure and was developed to follow the DSM-IV criteria [16] criteria for PTSD FC [14]. TES consists of 24 questions with four questions for criterion A (the stressor criterion), 17 questions for criterion B-D (re-experiencing, avoidance, and arousal) and one question for criterion E (time since the trauma) and F (the impact of the trauma on everyday life). Questions for criteria A-D follows by alternatives that gives 0–3 points, were the symptom sum score (criteria B-D) can go from 0 to 51 points, were a higher score indicate more symptoms of PTSD. TES has been evaluated in a Swedish context and considered clinically relevant. When used as a screening instrument for PTSD the recommended cut-off symptom score is ≥30 [33]. A preliminary validation of TES showed Cronbach’s α .92 for symptom sum score on a group of women meeting the criterion A, with a sensitivity of 94.4% and a specificity of 84% [33].

EPDS assesses symptoms of depression in the perinatal period. Respondents rate the intensity of symptoms during the previous 7 days [34]. It consists of 10 items, rated from 0 to 3, were higher scores indicates more depressive symptoms. When used in clinical settings, a cut-off point of ≥12 is often used [35]. EPDS has shown good validity when screening for post partum depression with good sensitivity (86%), specificity (78%), high standardised Cronbach’s α (.87) and split-half reliability (.88) [35].

SWLS assesses quality of life and well-being [36]. The five items are rated on a 7-point Likert-type scale ranging from 5 to 35, where a higher score indicate higher satisfaction with life. SWLS has good internal consistency, convergent validity and also good test-retest correlation (α = .87 and r = .82 respectively) [36, 37].

Finally, the WCQ is a self-report questionnaire, designed to assess and identify thoughts and actions individuals use to cope with a stressful event [38]. In this study a version with five of eight subscales were used (confrontive coping, self-controlling, seeking social support, escape-avoidance, and planful problem solving). The questionnaire consists of 33 items rated on a 4-point Likert scale ranging from 0 to 3 where a higher score indicates higher use of the coping method. The internal inconsistency is acceptable (α .61–.79) according to the manual [38].

### Sample size estimation and power

Information from a previous Swedish study [39] was used for power calculation. We estimated a conservative medium effect size (Cohen’s *d* = 0.5), *α* = .05, and a power of 0.8. A total sample of 130 participants was needed for between-subjects t-test contrasts and for categorical outcome measures with a medium effect size (*ω* = 0.3), *α* = .05, df = 1, and a power of 0.8 a total sample size of 88 participants was needed for *χ*^2^ test analysis.

### Data collection

Data were collected through questionnaires via the U-CARE web portal at four time points: (1) at baseline, about 8–16 weeks postpartum; (2) after step 1 of the intervention, 6 weeks after randomization; (3) after step 2 of the intervention, 14 weeks after randomization; (4) 1 year after randomization. Reminders for the questionnaires were sent regularly to participants. Participants who missed to fill out the questionnaires via the portal had paper-questionnaires sent home by post. Data were electronically stored on the U-CARE web portal and could only be retrieved by members of the research group.

### Ethics

The Regional Ethics Review Board in Uppsala approved the study (2012/495/1 and amendment 2016/11/16) and informed consent was obtained from all participants.

### Statistical analysis

Data were analysed using SPSS, version 26 [40]. Differences in demographic data and pre-treatment measures were analysed with independent measures using χ2 or t-test. For the continuous outcome data, a mixed-model repeated measures analysis (MMRM) was used. The assumptions for repeated measurements were met (e.g. Mauchly’s test of sphericity). Using a mixed-model analysis enabled the inclusion of all data since the analysis makes predictions based on available data, regardless of data missing at different time points, thus making it suitable for intent to treat (ITT) analysis. A MMRM model includes both fixed and random effects. Models were compared using the maximum likelihood method. We included two between levels (iCBT+TAU and TAU) and four within assessments (0, 6, 14 weeks and 1 year after randomization). After visual inspection of repeated measures, quadratic time effects were investigated where change was assessed as non-linear. Statistical analyses were first performed in an ITT principle, where each participant was analysed according to randomization. We then analysed the data per-protocol, where those who received iCBT were only included in the analysis if they received a minimum of three sessions of iCBT. We also planned for calculation of effect sizes for significant interaction effects between the groups at the last assessment (i.e., at 1 year follow-up), based on the estimated means, using Cohen’s d where 0.20 is considered a small effect, 0.50 a medium effect, and 0.80 a large effect [41]. Odds ratios regarding PTSD diagnosis were calculated between the groups. Exploratory sub-group analyses were performed in participants with solely negative birth experience, those who had an immediate caesarean section and those with a severe postpartum haemorrhage.

## Results

### Sample characteristics

The study sample consisted of 266 women recruited from the four sites (*n* = 199, *n* = 40, *n* = 20, and *n* = 7). The mean age was 31.8 years and 73% of the participants had a university degree and 27% had elementary school or high school as highest educational level (Table 1). We found no differences between groups (iCBT+TAU or TAU) regarding demographic and obstetric data or primary or secondary outcome measures at baseline. In the iCBT+TAU group 59 (44.7%) women were considered treatment completers (≥3 modules).

### Outcome analyses

#### Intention to treat (ITT) analysis

The ICBT intervention did not show superiority as both groups showed similar beneficial trajectories on several outcomes up to 1 year follow-up. For the primary outcome measures, we found significant time and quadratic time effects on the re-experience symptom scale of the TES (Table 4). This was due to a linear reduction from baseline to 14 weeks, but no change to 1 year follow up in both groups (Table 3). There was a significant main effect of time and quadratic time on the EPDS scale (Table 4). Both groups showed parallel decrease over time in reported depressive symptoms (Table 3).

**Table 3:** Estimated mean scores and SD at each time point, by group according to ITT

	Baseline		6 weeks		14 weeks		1 year	
	ICBT	TAU	ICBT	TAU	ICBT	TAU	ICBT	TAU
	*M* (*SD*)	*M* (*SD*)	*M* (*SD*)	*M* (*SD*)	*M* (*SD*)	*M* (*SD*)	*M* (*SD*)	*M* (*SD*)
TES								
B re-experience	9.02	9.62	8.66	9.20	8.29	8.75	8.30	8.45
	(2.74)	(3.28)	(2.67)	(3.21)	(2.58)	(3.11)	(2.22)	(2.75)
C avoidance	12.34	11.90	12.31	11.86	12.27	11.80	12.08	11.50
	(2.91)	(3.33)	(2.92)	(3.34)	(2.92)	(3.34)	(2.96)	(3.39)
D arousal	9.52	9.51	9.49	9.45	9.44	9.36	9.19	8.92
	(3.00)	(3.20)	(2.94)	(3.14)	(2.86)	(3.06)	(2.51)	(2.74)
EPDS	9.43	9.29	8.76	8.62	8.06	7.93	7.81	7.70
	(4.26)	(4.55)	(4.19)	(4.47)	(4.11)	(4.37)	(3.77)	(4.03)
SWLS	26.07	27.41	26.13	27.41	26.19	27.38	26.05	26.83
	(4.98)	(4.05)	(4.92)	(4.05)	(4.86)	(4.06)	(4.75)	(4.35)
WCQ								
Confrontive coping	5.87	5.78	5.87	5.80	5.88	5.83	5.89	5.97
	(1.43)	(1.46)	(1.43)	(1.47)	(1.44)	(1.47)	(1.46)	(1.50)
Self-controlling	7.69	7.11	7.80	7.22	7.95	7.37	8.64	8.05
	(1.63)	(1.74)	(1.63)	(1.75)	(1.64)	(1.76)	(1.68)	(1.80)
Seeking social support	7.40	7.88	7.23	7.64	7.11	7.42	7.97	7.83
	(2.29)	(2.47)	(2.20)	(2.42)	(2.09)	(2.37)	(1.96)	(2.48)
Escape-avoidance	7.31	6.50	7.20	6.51	7.04	6.52	6.33	6.56
	(2.66)	(2.75)	(2.68)	(2.78)	(2.71)	(2.83)	(2.93)	(3.13)
Planful probl. Solving	6.24	6.79	6.40	6.86	6.62	6.95	7.64	7.37
	(1.42)	(1.54)	(1.44)	(1.55)	(1.47)	(1.58)	(1.60)	(1.73)

*TES* Traumatic event scale, *EPDS* Edinburgh Postnatal Depression Scale, *WCQ* The Ways of coping questionnaire, *SWLS* The Satisfaction with Life Scale

**Table 4:** Time, interaction, and quadratic time effect from the MMRM analyses according to ITT

	Time	Group	Time X group
	*F* (*df*)	*F* (*df*)	*F* (*df*)
TES			
B re-experience^a^	8.96(1281.24)**	1.90(1290.20)	0.00(1169.23)
C avoidance	1.78(1170.79)	0.88(1290.27)	0.10(1170.17)
D arousal	0.23(1308.42)	0.00(1267.33)	0.32(1173.86)
EPDS^b^	10.02(1298.65)**	0.05(1267.08)	0.00(1169.23)
SWLS	0.00(1279.80)	4.46(1263.93)*	0.62(1158.96)
WCQ			
Seeking social support	1.88(1314.23)	1.20(1258.62)	0.76(1166.92)
Confrontive coping	0.34(1179.53)	0.07(1318.17)	0.12(1176.52)
Self-controlling	5.55(1186.86)**	2.16(1320.22)	0.00(1183.44)
Escape-avoidance	0.02(1168.88)	2.80(1293.83)	2.34(1167.46)
Planful problem solving	1.83(1186.61)	1.97(1322.87)	1.56(1183.24)

*TES* Traumatic event scale, *EPDS* Edinburgh Postnatal Depression Scale, *WCQ* The Ways of coping questionnaire, *SWLS* The Satisfaction with Life Scale.

^a^ Quadratic Time was a significant predictor *F* (*df*) = 5.0*, (1268.20).

^b^ Quadratic Time was a significant predictor *F* (*df*) = 6.45* (1273.13).

* *p* < .05, ** *p* < .01

For the secondary outcome measures, we found a significant main effect of treatment on the SWLS scale (Table 4) due to higher initial reported satisfaction of life in the TAU group followed by a small decrease over time while the iCBT+TAU group reported no change (Table 3). The WCQ subscale *Self-controlling* showed a significant main effect of time (Table 4) with a parallel increase over time in both groups (Table 3). No effect size calculations were made, due to non-significant interaction effects (see Table 3).

### Completer analyses

For the primary outcome measures, both the re-experience symptom scale of the TES and the EPDS showed significant time and quadratic time effects (Table 6) due to similar trajectories as reported in the ITT analyses (Table 5).

**Table 5:** Estimated mean scores and (SD) at each time point, by group for treatment completers

	Pre		6 weeks		14 weeks		1 year	
	ICBT	TAU	ICBT	TAU	ICBT	TAU	ICBT	TAU
	*M* (*SD*)	*M* (*SD*)	*M* (*SD*)	*M* (*SD*)	*M* (*SD*)	*M* (*SD*)	*M* (*SD*)	*M* (*SD*)
TES								
B re-experience	9.13	9.64	8.72	9.17	8.29	8.68	8.42	8.46
	(2.79)	(3.29)	(2.72)	(3.22)	(2.64)	(3.14)	(2.41)	(2.81)
C avoidance	13.15	11.90	13.12	11.86	13.09	11.80	12.91	11.50
	(3.27)	(3.34)	(3.28)	(3.35)	(3.29)	(3.36)	(3.36)	(3.41)
D arousal	9.69	9.54	9.64	9.42	9.59	9.29	9.64	8.93
	(3.11)	(3.19)	(3.05)	(3.14)	(2.97)	(3.06)	(2.65)	(2.74)
EPDS	9.96	9.34	9.16	8.57	8.32	7.78	8.01	7.71
	(4.79)	(4.58)	(4.72)	(4.50)	(4.63)	(4.40)	(4.27)	(4.01)
SWLS	26.17	27.44	26.18	27.38	26.20	27.28	26.26	26.84
	(4.99)	(3.98)	(4.98)	(4.02)	(4.99)	(4.07)	(5.22)	(4.55)
WCQ								
Confrontive coping	5.61	5.78	5.63	5.80	5.64	5.83	5.73	5.97
	(1.53)	(1.49)	(1.55)	(1.50)	(1.57)	(1.52)	(1.70)	(1.61)
Self-controlling	7.45	7.11	7.55	7.22	7.69	7.37	8.36	8.05
	(1.72)	(1.73)	(1.72)	(1.73)	(1.74)	(1.74)	(1.80)	(1.79)
Seeking social support	7.77	7.94	7.43	7.59	7.11	7.26	7.75	7.86
	(2.33)	(2.50)	(2.21)	(2.44)	(2.08)	(2.38)	(2.03)	(2.49)
Escape-avoidance	7.22	6.50	7.07	6.51	6.87	6.52	5.93	6.55
	(2.94)	(2.84)	(2.96)	(2.87)	(3.00)	(2.92)	(3.24)	(3.17)
Planful probl. Solving	6.10	6.79	6.29	6.86	6.55	6.95	6.55	6.95
	(1.58)	(1.57)	(1.60)	(1.59)	(1.62)	(1.61)	(1.62)	(1.61)

*TES* Traumatic event scale, *EPDS* Edinburgh Postnatal Depression Scale, *WCQ* The Ways of coping questionnaire, *SWLS* The Satisfaction with Life Scale

For the secondary outcome measures, there was a significant quadratic time effect on the WCQ subscale *Seeking social support* (Table 6) due to parallel change over time in both groups with linear decrease from baseline to 14 weeks followed by increase at 1 year follow up (Table 5). There was a significant time effect (Table 6) on the subscale *Self-controlling* of the WCQ following the same pattern as mentioned in the ITT analysis (Table 5). No effect size calculations were made, due to non-significant interaction effects (see Table 5).

**Table 6:** Time, interaction, and quadratic time effect from the MMRM analyses according to treatment completers

	Time	Group	Time X group
	*F* (*df*)	*F* (*df*)	*F* (*df*)
TES			
B re-experience ^a^	17.46(1250.05)***	0.87(1189.26)	0.95(1140.33)
C avoidance	1.64(1146.30)	4.18(1206.90)*	0.09(1146.18)
D arousal	0.63(1251.22)	0.07(1192.28)	1.10(1143.41)
EPDS ^b^	11.30(1243.05)**	0.52(1192.62)	0.15(1141.53)
SWLS	1.70(1156.80)	4.63(1273.49)*	0.61(1157.71)
WCQ			
Seeking social support ^c^	3.59(1250.52)	0.09(1182.21)	0.01(1136.70)
Confrontive coping	0.35(1150,28)	0.18(1230.18)	0.02(1150.71)
Self-controlling	5.38(1156.98)*	0.46(1227.71)	0.01(1157.45)
Escape-avoidance	0.02(1141.92)	1.41(1213.28)	3.29(1142.14)
Planful problem solving	1.84(1161.94)	2.02(1236.67)	2.07(1162.71)

*TES* Traumatic event scale, *EPDS* Edinburgh Postnatal Depression Scale, *SWLS* The Satisfaction with Life Scale, *WCQ* The Ways of coping questionnaire.

^a^ Quadratic time was a significant predictor: *F* (*df*) = 10.86*** (1227.483).

^b^ Quadratic time was a significant predictor: *F* (*df*) = 7.76**, (1225.98).

^c^ Quadratic time was a significant predictor: *F* (*df*) = 3.92*, (1227.14).

* *p* < .05, ** *p* < .01, ***p < .001

### Subgroup analyses

Exploratory analyses were performed including either those with low rating/negative birth experience (*n* = 195), or immediate caesarean section (*n* = 39), or severe haemorrhage (*n* = 32). Among those who were included due to low rating/negative birth experience we found a significant time (*p* < .001) and a quadratic time effect (*p* = .001) on the re-experience symptom scale on the TES and a significant time effect (*p* < .001) due to improvement on the arousal scale of the TES. There were no other significant time, group or interaction effects among the subgroups immediate caesarean section or haemorrhage on the TES subscales. There was a significant time (*p* = .001) and quadratic time effect (*p* = .013) on the EPDS scale (lower depression scores) among those included due to rating of their birth experience and no other significant effects were found among the other two subgroups. The WCQ subscale *Self-controlling* showed a significant time effect (*p* = .023; increased rating) among those included due to rating of their birth experience. No other significant effects among the subgroups were found on the secondary outcome measures.

### PTSD FC diagnosis according to the TES

There was no association between group and PTSD diagnosis (yes / no) at baseline assessment (χ^2^(1254) = 2.44, *p* = .12). Odds ratios indicate the risk of having no PTSD diagnosis in the iCBT group compared with the TAU group. Odds ratio showed no benefit for the iCBT group at 6 weeks (OR = 1.94 [95% CI: 0.49–7.94]), at 14 weeks (OR = 2.43 [95% CI: 0.44–13.36]), or at 1 year follow up (OR = 0.49 [95% CI: 0.11–2.31]). Only a few women fulfilled the criteria for PTSD FC after iCBT step 1, which precluded meaningful analyses of the result of iCBT step 2.

## Discussion

In this study, we evaluated the efficacy of an internet-based CBT-intervention for women with negative birth experiences and/or at risk for PTSD FC aimed at reducing symptoms of posttraumatic stress, depressive symptoms and increase quality of life and ability to cope.

### Principal findings

We could not demonstrate any benefit of iCBT +TAU compared to TAU on PTSD FC at 6 weeks, at 14 weeks or at 1 year follow up. However, both groups (ICBT+TAU and TAU) in the ITT- and completer-analyses reported reduced symptoms on the subscale re-experiencing on TES over time (baseline to 14 weeks). The subscale includes symptoms of re-experiences of the trauma like flashbacks, dreams, intrusive memories, and the feeling of reliving the negative experiences. The majority of individuals who have been exposed to a traumatic event recover spontaneously by natural recovery or resilience [42]. Reduction of symptoms of re-experiences of the traumatic event could therefore be explained by a natural recovery for both the control and the intervention group. In line with these results, we found no significant benefit for the iCBT group regarding not meeting the criteria for PTSD at the three assessment points after end of treatment.

Results similar to ours were seen in a randomized controlled trial aimed at evaluating the feasibility of iCBT for women with PTSD following childbirth [29]. After 8 weeks of iCBT significant reductions of PTSD symptoms were found in the treatment group as well as in the control group, and the treatment group showed the same reduction of PTSD symptoms as the control group. Although the RCT of Nieminen at al [29]. differed from ours in several ways, all participants reported posttraumatic stress symptoms related to their delivery more than 3 months postpartum. Women were included when they had a score on the Traumatic Event Scale (TES) over 30 points. Participants were divided into a group that received immediate treatment and a group put on a waiting list (control group), which would receive treatment 5 months later. The treatment group constituted of 24 women and the control group 19 women. The treatment consisted of eight online modules, each with a therapy track and a homework track.

So far, only a few RCT’s have evaluated the efficacy of different psychological treatments for women with negative birth experiences and/or PTSD following childbirth [28, 43]. One RCT on CBT for this population has been conducted. The study had a small sample size and did not achieve the number of participants for a power of 80% [29]. Therefore, we know very little about suitable interventions and how this population engage and commit to iCBT and similar interventions. In a review that aimed to assess the available treatment options for PTSD following childbirth, six studies met the inclusion criteria [43]. The authors concluded that debriefing, CBT and EMDR are available as treatment for PTSD following childbirth and the authors also pointed out the need of more RCT’s before drawing conclusion about the effectiveness of these treatments.

The inclusion criteria (the cut off for negative birth experience on the Likert scale, immediate caesarean section, and severe postpartum haemorrhage) could have been over-inclusive and may not always correlate to risk of developing PTSD FC. It is well known that the subjective experience of the birth as negative is a risk factor for PTSD FC, but only a few women in this study fulfilled the criteria for PTSD according to TES at baseline. In a Swedish RCT, the effect of iCBT for women with PTSD following childbirth was evaluated [29]. Participants were recruited via nationwide invitation and were thereafter extensively evaluated and diagnosed with validated measurements before entering the study. This way of recruiting participants is more time consuming but also more likely to capture motivated participants with a proper diagnosis for the intervention compared to our study.

There was also a significant main effect over time on the EPDS scale where depressive symptoms decreased over time in both groups. This result can also be explained by a natural recovery in most women. The same pattern was seen in the study of Nieminen et al. [29] where participants in both control- and treatment group had positive effects on depressive symptoms.

SWLS is a short item instrument designed to measure global cognitive judgements of satisfaction with one’s life. In the ITT- analysis, the TAU-group initially reported a higher satisfaction of life compared to the iCBT+TAU group, but the differences diminished over time due to a decrease in the TAU-group and no changes in the iCBT+TAU group. The time after birth can be emotionally challenging for the mother. Mood swings due to the new situation probably affect the mother’s estimation of her life satisfaction. We cannot explain why the TAU group initially reported a higher satisfaction of life comparing to the iCBT+TAU-group. In the RCT of Nieminen et al. [29] quality of life was improved in the treatment group with a small to moderate effect size, but was not changed in the control group.

In the ITT- and completer analyses of WCQ both groups showed significant main effects of time on the subscale Self-controlling due to reported increase in this coping strategy over time. The subscale Self-controlling includes efforts to regulate one’s feelings and actions [38]. In the completer-analysis of WCQ it was also a significant quadratic time effect on the subscale Seeking social support, where both groups changed over time by first decreasing from baseline to 14 weeks but increased at 1 year follow up. The subscale seeking social support includes efforts to seek informational support, tangible support, and emotional support [38]. In several cultures it is normal and accepted for new parents to spend the first time alone with the baby, before inviting other people to their home. Therefore, the results of a decreased effect on Seeking social support followed by increase during the first weeks with the new-born seem normal in the western context.

In our exploratory sub-analyses, we found significant time and quadratic time effect on TES (the subscale re-experiencing) and significant time effect on TES (the subscale arousal) for the group with a negative birth experience. For the same group we also found a significant time and quadratic time effect on EPDS and a significant time effect on the WCQ-subscale *Self-controlling*. The subgroups with immediate caesarean section and severe haemorrhage included few participants, which limits the interpretation of the results.

The majority of the eligible women declined participation in the trial. Further, dropout-rates were generally high, both before and after randomization. Reasons and predictors for non-participation and dropout can be analysed from different perspectives. In a meta-analysis from 2010, three broad categories of predictors for dropout in internet interventions were identified: sociodemographic factors and contextual variables, psychological problems, and treatment-related variables [44]. In our study, one explanation for non-participation could be that our inclusion criteria (low rating of birth experience, immediate caesarean section or severe postpartum haemorrhage) were over-inclusive and did not fully correlate with a negative birth experience.

Only a handful of the randomized women reached the inclusion criteria for iCBT part 2, which precluded meaningful analyses. However, dropout in internet interventions is also generally high, especially in self-help programmes with little or no support [45]. More narrow inclusion criteria might have captured a more motivated and afflicted population. Also, regular weekly feedback and support from the psychologist instead of support on demand could have increased the treatment-adherence. On the other hand, self-help is a more cost-effective alternative for the provider than guided iCBT. Another explanation for non-participation or dropout could be that some women were so affected by the childbirth that they totally avoided participation in the study. Others might have dropped out because of by lack of time, tiredness, or exhaustiveness due to a demanding post-partum period. More research is needed to better understand how and when to offer professional support to women with negative birth experiences. Overall, the healthcare system needs to find effective ways to identify women in need of support by screening, asking questions and encourage women to talk about their symptoms.

### Strengths and limitations

This is the first iCBT were women with negative birth experiences and/or posttraumatic stress were clinically recruited. Clinical recruitment increases the probability for a representative sample and indicates how the intervention would work in a natural clinical context. There are several other strengths in this study, the randomised controlled design, the sample size, and the equivalence between the groups at baseline, all of which are important factors for the validity [46]. An important advantage is the internet-format, which has not only a number of general benefits, but also ensures a certain degree of anonymity, online interventions pose a useful alternative and might reduce the barriers of help-seeking. We believe that iCBT is extra suitable for postnatal women, the intervention offers flexibility, and the participant can work with the material from home and at any preferred time.

This study also has limitations. We did not screen for PTSD, depression or other psychiatric disorders and the wide inclusion criteria are giving room for sample heterogeneity. The generalizability of our findings to the population of women with negative birth experiences and/or at risk for PTSD FC in a clinical setting is limited owing to the Internet-based setting of the treatment and our recruitment strategy. Factors such as accessibility to the internet, language, education, or interest in technology can have restricted the range of women who participated in our trial. We delivered a standardized manualized approach for all women. Using this approach, we cannot say whether all women benefited equally from the different steps and/or if some parts were particularly helpful. In this study we had no information about the specific content of the TAU, therefore we cannot know if or how TAU had an impact on the participant’s symptoms. We chose TAU as control condition, which does not allow us to control for an active treatment intervention. Future studies should therefore implement comparator conditions such as an attention placebo group or some other active treatment aimed at the symptoms presented in this population.

Another methodological limitation was the poor treatment adherence and participants being lost to follow up. Retention at follow-up dropped considerably and explanations could be that the participants’ motivation dropped after a while. Some individuals may find it demanding to participate in a clinical trial, with all the interventions and follow-ups. It is also well known that internet interventions with regular guiding generally are more effective than non-guided interventions [47]. In our study, participants could contact a therapist on demand, but in the RCT of Nieminen et al. [29] the intervention included individual feedback every week by a personal therapist, which could explain a higher adherence.

The psychological health of the mother and/or a busy time being a new mother, could also have affected the level of motivation and adherence. Many women with a negative birth experience have a natural psychological recovery, which also could explain dropout in this study.

## Conclusions

In conclusion, this study outlines the development process and feasibility of an internet-based intervention for women with negative birth experiences and/or at risk for PTSD FC. We could not demonstrate any clear effect of the intervention, but changes over time in both groups indicate some level of natural recovery from birth related trauma. Inclusion criteria have to be stricter in order to evaluate the efficacy of the intervention.

### Future directions

The challenge in future research will be to develop interventions that are both well accepted and effective in supporting women with negative birth experiences and/or at risk for PTSD FC. The next step might be to apply more narrow inclusion criteria in order to capture a more motivated population in need of psychological support.

## Data Availability

The datasets used and/or analysed during the current study are available from the corresponding author on reasonable request.

## Abbreviations

CBT: Cognitive behavior therapy
ICBT: Internet based cognitive behavior therapy
ITT: Intention-to-treat
TAU: Treatment as usual
RCT: Randomized controlled trial
